# Reversing Anticoagulants: Influence of Anticoagulation Stewardship and Key Considerations for Optimizing Outcomes

**DOI:** 10.1177/10600280251356019

**Published:** 2025-08-03

**Authors:** William E. Dager, Megan A. Rech, Brian W. Gilbert

**Affiliations:** 1 University of California, Davis Health System, Sacramento, CA, USA; 2 Department of Veterans Affairs, Center of Innovation for Complex Chronic Healthcare, Edward Hines Jr VA Hospital, Hines, IL, USA; 3 Department of Pharmacy, Wesley Medical Center, Wichita, KS, USA

**Keywords:** anticoagulation, antifactor Xa, bleeding, stewardship, reversal, clinical pharmacy, andexanet, prothrombin complex concentrates, direct oral anticoagulants, outcomes

## Abstract

The reversal of anticoagulants can be a complex process with limited data describing the optimal overall approach beyond a specific reversal agent. Recent advances in anticoagulation stewardship have created opportunities to standardize and optimize the reversal of anticoagulants, especially in urgent life-threatening bleeding events. This article explores how pharmacists provided anticoagulation stewardship activities positively impact outcomes related to urgent anticoagulation reversal.

## Introduction

Antithrombotic therapy, including anticoagulants and antiplatelet agents, is a cornerstone of medical care across various disease states, presenting multiple challenges in a personalized medicine approach to optimizing overall outcomes. Precision-based anticoagulant therapy is especially challenging and a common consideration in the critically ill.^1^ A key aspect of antithrombotic therapy is mitigating the risk for adverse events, such as bleeding or recurrent thromboembolism, which are associated with a high rate of preventable admissions to the emergency department (ED).^2^ Given these concerns, the concept of antithrombotic stewardship has been an evolving focus in recent years. The Joint Commission on Accreditation recognizes the importance of anticoagulation reversal and includes it as a specific safety goal.^3^ Randomized controlled trials and more stringent analyses have sought to answer many key questions on the best approaches to reversing anticoagulants.^4-6^ Guidelines and expert opinion guidance statements provide insights from the current evidence for management approaches that can incorporate the key drivers for optimal outcomes. However, limitations regarding the populations studied, delays created by the focused inclusion criteria and consent process can impact the population studied and prolong the time to reversal administration. In addition, regional differences in practice and the caregivers involved can influence the best overall approach to reversing these agents. The critically ill presenting with life-threatening bleeding and limited time (e.g. less than 1 hour) to stabilize the situation would be excluded from much of the evidence gathered in addition to challenges created with heterogenous populations requiring anticoagulation reversal. This can make it difficult to elucidate a single reversal agent and approach for all situations. Results from clinical trials and guidance recommendations may not be generalizable across all populations, health care settings with limited resources or situations where timing, bleeding severity, ability to provide selected reversal agents and operational considerations vary in real world practical settings where a precision-based multidisciplinary approach should be a primary consideration.^1^

## Multidisciplinary Anticoagulation Stewardship Influencing Reversal

Multidisciplinary anticoagulation stewardship and increasing knowledge of appropriate patient selection and reversal targets have improved management and outcomes independent of the hemostatic agent utilized.^7,8^ Table 1 provides examples of where anticoagulation stewardship programs provide insights to maximize desired outcomes and minimize adverse effects which include challenging traditional dosing schemes of commonly utilized reversal agents.^9^ Management goals may consider optimizing dosing to deliver the minimum necessary dose to achieve outcomes while minimizing the potential adverse effects such as thromboembolism or prolonged reversal delaying re-initiation of anticoagulation. Examples include the use of oral (PO) or intravenous (IV) vitamin K to reverse warfarin where historically, 10 mg frequently in the past given by subcutaneous administration for 3 daily doses, has been shown to take longer and less effective than the IV or PO route. This has now been reduced to much lower single doses recognizing that full effects to completely reverse the international normalized ratio (INR) can occur with 2 to 3 mg IV.^10^ In selected situations, vitamin K doses as low as 0.25 mg IV created partial and shorter reversal periods (potentially desired in patients with mechanical heart valves or left ventricular assist devices).^11^ Other stewardship driven examples include reduced dose of prothrombin complex concentrate (PCC), which used to start as high as 5000 factor IX units, where in unique settings, much lower single doses may be considered (e.g. 2,000 units to as low as 500 units) to prevent sustained periods of reversal.^12,13^ In addition, single set PCC dosing independent of the INR can reduced time to administration and avoid delays associated with initial INR obtainment and reporting when reversing warfarin.^7,14^ The first published case of successful reversal of a direct oral anticoagulant-related massive life-threatening bleeding event using an activated PCC was driven by pharmacists involved in anticoagulation and hemostasis stewardship.^15^ Separately, this same stewardship program dispelled information from clinical trials that omitted the key driving factor of tissue re-bound influencing how much dabigatran is removed during hemodialysis.^16,17^ These are some of many examples of such pharmacist related and innovative stewardship practices.

**Table 1.** Considerations to Expedite Time to Reversal Agent Administration.

Activity	Comment
Determine in advance with multidisciplinary input the approach to reversing specific anticoagulants and how this will be done.	Determine what reversal agents are on formulary, and when they would be used. Consider breaking the plan into urgent (need to reverse immediately), semiurgent (need to reverse within an hour or so) and nonurgent (watch and keep reversal in mind should it be needed). Include what labs should be drawn and when. Summarize in a decision support process. Predetermined nonweight-based dosing (e.g. 2000 units) may be a consideration instead of weight-based dosing (units/kg) or dependence on the INR being reported (warfarin) first to determine the dose prior to initiating therapy. Develop pathways to determine what anticoagulant is present if no history is available.
Develop anticoagulant agent class specific order sets	Include requests for any information needed to fulfill policy restrictions, pertinent allergy (heparin) information, and any needed baseline labs (ordered as a priority) or labs for subsequent assessments. Provide specifics on how to dose the reversal agents including route, dose and rate of administration
Stock agents as close to the point of care if possible	Selected products may not require compounding in a central sterile environment. Such agents may be stocked in the automated drug dispensing cabinet near the patient’s bed. A pharmacist or designated nurse may have the capabilities of removing from stock and have instructions on how to mix and prepare for administration including the rate.
Make sure any pharmacy admixture can be done without delay	When preparation in a sterile environment is necessary, provide clarity on where this will be done, how to contact the site and keep them informed of the patient’s location to deliver the reversal agent. Provide instructions in advance for the IV Admixture staff on how to expedite the process as urgent and simplified instructions on preparation to avoid any delays. Consider laminated cards with the agents on how to do the admixture to limit any time spent understanding how to prepare infrequent requests. Move the process forward ahead of less urgent requests. Expedite prompt delivery of the agent to the patient’s beside. A designated pharmacist or technician may need to be identified to immediately deliver the drug to the bedside.
Verify orders promptly and obtain the necessary label if required for scanning prior to administration	Limit any delay in verification. Order sets should be developed to provide any necessary information that may be needed to know the request is safe and meets set policy. If 2 pharmacists are present at the bedside, one can process and verify the order while the other is expediting preparation.
Make sure any infusion pump libraries are programed for the reversal agent to be infused	For all reversal agents including concentration, rate of infusion should be preprogramed into the pump library and easy for the nurse to access and program in.
Establish seamless communication	Communication between ordering providers, pharmacy and nursing should be an easy process. Each party needs to provide direct access to limit any delays in clarifying any confusion or recommendations that need to be resolved.

Abbreviation: INR, international normalized ratio; IV, intravenous.

Other anticoagulant stewardship activities include education to both clinicians and patients, agent selection, and dosing regimen modifications, including any follow-up assessments (Table 1). This can include plans for restarting anticoagulation at either prophylaxis or treatment levels that can minimize potential post reversal thromboembolism risks and include a focus on transition of care, all potentially leading to shortened duration of hospitalization and related cost savings.^18^ From a systems perspective, anticoagulation stewardship programs undertake prospective or retrospective medication reviews (e.g. guidelines, checklist or protocol-driven reviews, electronic interventions) to identify gaps and potential areas for improvement, development of management polices; and expedite initial and longer-term management processes that include reversal therapies. Examples may include bedside presence to facilitate management as events unfold, accessibility for all engaged parties to rapidly communicate, the development of specific order sets and decision support tools to guide the reversal process and minimize any delays.

Pharmacists play a critical role in anticoagulation stewardship through involvement in specialized teams such as Pulmonary Embolism Response Teams (PERT) and stroke response teams expediting time sensitive treatments. In addition, the implementation of decision support tools, including standardized order sets, has streamlined the process from patient evaluation to the administration of hemostatic agents, improving both efficiency and patient outcomes.^8,19-21^ Similar to door-to-needle times in acute ischemic stroke (AIS), the time to anticoagulant reversal in intracerebral hemorrhage (ICH) has been recognized as a metric that should be targeted. The 2024 American Heart Association/American Stroke Association Performance and Quality Measures for Spontaneous ICH recommend targeting anticoagulant reversal within 60 to 90 minutes of diagnosis.^22^ European emergency clinicians have also identified the importance of time immediate therapy, but do not discuss the logistics and practitioners involved.^23^ The time from door to treatment can be prolonged, ranging from less than 30 minutes to several hours.^24^ A recent study of U.S. hospitals participating in Get With The Guidelines – Stroke found that earlier anticoagulation reversal was associated with improved survival for patients with ICH.^24^

Pharmacists are an important proponent in assisting in utilization of the available agents on formulary to reduce the time to reversal agent administration and facilitating blood pressure management in the ICH cohort. In the setting of an ICH, reduced mortality and earlier discharge from the hospital was associated with reversal implementation within 60 minutes and remains a targeted outcome of interest by the American Heart Association.^24^ Several retrospective studies have demonstrated that pharmacists intervention reduces time to hemostatic agent administration in ICH.^8,19-21^ These studies have noted that having a pharmacist in the ED for management of ICH reduces the overall time for reversal by 36 to 140 minutes (206 to 60 minutes, 70 to 35 minutes, 85 to 50 minutes and 60 to 21 minutes). In addition, it has been demonstrated that faster door-to-treatment times were associated with shorter length of intensive care unit (ICU) and hospital length of stay.^8,24^ The recently published CODE ICH recommendations include reversal of anticoagulants and blood pressure optimization within 60 minutes to reduce hematoma expansion. Both activities can be facilitated and expedited by a pharmacist on a multidisciplinary team to meet all the patient’s overall management needs.^25^ As post reversal thrombosis is a concern, facilitating a plan for re-initiation of anticoagulation, especially as patients transfer services, is an additional benefit provided by stewardship activities.

## Improving the Anticoagulation Reversal Process

Identifying ways to improve timely intervention can be multifactorial. Beyond the initial assessment and decision on what therapy to implement is the logistics of getting the chosen therapy to the bedside and administered. This is one place where pharmacist involvement at the point of care can facilitate the order entry, verification, and logistically expedited provision of the product at the bedside. Prothrombin complex concentrates can be stored in automated dispensing machines close to the point of care where they can be removed, reconstituted and administered promptly. For intravenous or specific unit dose reversal products in the United States, admixture requirements in a centralized sterile environment involving personnel less familiar with the urgency of the situation can create delays to administration. This can be driven by the process of preparing the doses once the order is verified, and compounding at a separate location and distance from the patient in addition to challenges of knowing where the patient is located and arranging delivery. If such agents are chosen, stewardship can minimize potential delays by facilitating the preparation process to avoid any delays in getting the therapy administered. Costly reversal therapies may have additional barriers beyond centralized compounding including formulary restrictions that need to be addressed prior to dispensing that can be facilitated and expedited with stewardship involvement. An additional challenge includes reaching providers to clarify any concerns and importance of timing along with other “priority” tasks. Historically, rural hospitals may have limited availability of concentrated clotting factors, or even less, a specific reversal agent outside of vitamin K and may require transferring the patient to a higher care setting to receive the necessary management.^26^

## Potential Impacts of Pharmacist Driven Antithrombosis and Hemostasis Stewardship

Pharmacist-related stewardship can improve the safe use and optimize outcomes with anticoagulants in multiple ways related to bleeding and reversal (Table 1). Prospective stewardship in one study demonstrated a 44% reduction in direct oral anticaogulant (DOAC) related medication errors, and medication cost.^27^ Assessing for any potential root causes of the bleeding may help prevent recurrent events by eliminating them or revising therapy. This can include potential drug interactions or disease states reducing the anticoagulant’s elimination that creates excessive serum concentrations, poor understanding on how the medications should be taken and preventing activities or situations placing the patient at risk for a traumatic event. Additional examples of prospective stewardship activities include the ability to reverse the effects of bivalirudin with low dose actived recombinant factor VII during open heart surgery, as previously mentioned using lower doses of Vitamin K or PCC for partial reversal, or adding vitamin K to PCC administration to reverse warfarin to prevent rebound in the INR and desire for prolonged reversal.^13,28^

Recently, the use of andexanet alpha (AA) was compared to usual care for the reversal of anti-Xa oral anticoagulants in the ICH cohort.^5^ Overall, it was noted that AA resulted in better control of 12-hour hematoma expansion compared to usual care (of which 86% was PCC), but was associated with a higher incidence of thrombotic events, especially concerning arterial locations including AIS. Vigilance in re-initiating anticoagulation therapy was apparently not a focus of the trial. Approximately three-quarters of the 452 patients enrolled in the study were managed in Europe, with the remainder treated in North America. In North America, usual care showed a nonstatistical trend toward better efficacy. In addition, the median time from hospital presentation to treatment was slightly faster with AA (2.1 hours) compared to usual care (2.3 hours). Although either product may be equivalent overall in long term outcomes, the resulting difference may have been influenced by stewardship and the operational process of reversal that reaches patients faster as much as the agent and dose selected.^29^ Although unclear, patients in systems with rapid responses, attention to re-initiating anticoagulation and addressing root causes for the bleeding may not be adequately represented in randomized consent based clinical trials. Another larger retrospective data set on an ICH cohort examined the efficacy of reversing anti-Xa DOACs. Among the 663 patients studied by neurocritical care focused pharmacists primarily in North America, efficacy was observed in 433 patients where treatment was administered approximately 2.6 hours after admission.^30^ Hemostatic efficacy was 88.1% with aPCC (median dose 26.7 units/kg) and 79.4% with inactivated 4-factor PCC (median dose 43.8 units/kg), both of which were higher than the overall efficacy rates of 53.1% with usual care and 67% with AA in the ANNEXA-I trial.^5,29^ It should be noted that the subgroup analysis of North American data from ANNEXA-I is currently not available, and there is a potential bias regarding resources and selection of the group managed by neurocritical care pharmacists, although they included Glascow Coma Scores below 7 (9.2% of patients), which was an exclusion in the ANNEXA-I trial. In addition, the reported times to reversal administration was over 2 hours, creating a concern that should be explored in more depth that such delays may have had a notable impact outcomes.

## Summary

In summary, these data explore key considerations relative to the impact of pharmacists involved in multidisciplinary anticoagulation stewardship for the reversal of anticoagulation stewardship. The closer and sooner care can be provided to the patient that incorporates awareness of the needs of all involved parties is core to the process. Rapid assessment, order placement and verification that can be facilitated by use of specific order sets and decision support tools to reduce times for preparation and administration are part of the key components to shorten the time to intervention regardless of what agent is chosen.

## Conclusion

Anticoagulation stewardship provides many opportunities to improve the management of antithrombotic therapy, including the reversal of anticoagulants. The utilization of anticoagulation stewardship and involvement of advanced clinical care pharmacists, which is more common and established in North America, has demonstrated a positive impact on improving care. Such practitioners also provide additional support in patients requiring reversal by re-initiating anticoagulation therapy, stopping anticoagulants no longer necessary to give and transitions of care. Utilizing such services close to the point of care, especially in critical bleeding can lead to improved outcomes. Assessments and studies exploring the optimal way to prevent and manage bleeding events in the setting of anticoagulation therapy must consider all the potential drivers for efficacy.
